# Quality of follow-up care for premature infants in primary health care: A cross-sectional study from nurses' perspective in Paraná state, Brazil

**DOI:** 10.1371/journal.pone.0351395

**Published:** 2026-06-10

**Authors:** Ivaneliza Simionato de Assis, Geisyelli Alderete, Regiane Bezerra Campos, Marcos Augusto Moraes Arcoverde, Debora Falleiros de Mello, Adriana Zilly, Rosane Meire Munhak Silva

**Affiliations:** 1 Postgraduate Program in Public Health in Border Region, Universidade Estadual do Oeste do Paraná, Foz do Iguaçu, Paraná, Brazil; 2 Centro Universitário Dinâmica das Cataratas, Foz do Iguaçu, Paraná, Brazil; 3 Department of Maternal-Child Nursing and Public Health, Ribeirão Preto College of Nursing, Universidade de São Paulo, Ribeirão Preto, São Paulo, Brazil; Federal University of Ceara, BRAZIL

## Abstract

**Background:**

Prematurity affects approximately 10% of births worldwide and represents the leading cause of mortality in children under five years of age. The considerable variability in follow-up programs for premature infants internationally indicates the need for standardized protocols. In Brazil, the discontinuity of follow-up care for children after hospital discharge constitutes a significant weakness, particularly in regions with socioeconomic disparities. Despite the essential role of Primary Health Care, there are gaps in evaluating the quality of follow-up care for premature infants from the perspective of health professionals, particularly nurses. This study aimed to the perceived quality of follow-up care for premature children in Primary Health Care from the perspective of nurses working in Paraná state, Brazil.

**Methods:**

Cross-sectional, descriptive, and analytical study with a quantitative approach, conducted with 463 nurses working in Primary Health Care between 2024 and 2025 in Brazil. The “Qualipreterm” instrument was used to evaluate five quality domains: hospital discharge planning, home follow-up, child health follow-up, integration between services, and family support. Descriptive analyses, chi-square tests, and spatial distribution description through thematic maps were performed.

**Results:**

From the nurses’ perspective, Domain I (hospital discharge planning) showed 58.9% of evaluations classified as inadequate. Domain V (family support) demonstrated the best performance, with 58.5% of evaluations classified as good and 3.5% as excellent. Domains II, III, and IV showed a predominance of regular classification (56.4% to 59.8%). Regional analysis revealed heterogeneity among the macro-regions studied, with some showing greater consistency in quality while others faced greater challenges in hospital discharge planning.

**Conclusions:**

The study reveals weaknesses in the perceived quality of follow-up care for premature infants in Primary Health Care, from the perspective of nurses, indicating the need for implementation of standardized protocols that promote longitudinal care, increased coordination strategies between healthcare network service points, and strengthening of continuing education for healthcare professionals.

## Introduction

Prematurity represents one of the major challenges in maternal and child health worldwide, being responsible for high neonatal morbidity and mortality and having a significant impact on global public health. According to the World Health Organization (WHO), preterm birth, defined as birth occurring before 37 weeks of gestation, affects approximately 10% of global births, constituting the leading cause of mortality in children under five years of age [1]. In Brazil, particularly in regions with socioeconomic and structural disparities, adequate care for premature newborns is essential to minimize sequelae and promote healthy development [2,3].

Follow-up care for preterm infants is an international challenge that requires continuous and integrated follow-up programs in Primary Health Care (PHC). A study conducted in Europe demonstrated variation in follow-up programs for very preterm children, highlighting the need for standardized protocols to ensure developmental monitoring and early identification of complications [4]. Furthermore, international models of care for high-risk premature infants emphasize the importance of a multidisciplinary approach and the use of technologies to enable continuity of care, reducing morbidity and mortality [5].

The initial hospitalization of these newborns requires intensive care, the length of which may be associated with factors such as birth weight, gestational age, and secondary comorbidities [6,7]. However, hospital discharge does not represent the end of the care process, as the transition from hospital to home requires continued follow-up in PHC services, where longitudinal care and support are essential for early detection of developmental problems and ensuring timely interventions [8,9].

The transition from hospital care to home is a critical moment for maintaining quality and safety in the care of premature infants. Strategies such as home visits and telemonitoring are important tools that maintain the bond between families and healthcare professionals, strengthening parental support and facilitating communication [8]. These practices underscore the need for constant evaluation of the quality of PHC services in post-neonatal and infant discharge [10].

Discontinuity of follow-up is one of the main weaknesses faced by premature infants after hospital discharge, especially in areas with social and geographic barriers, such as border regions [11,12]. Studies emphasize the importance of efforts to identify and direct vulnerable populations during initial hospitalization, before premature infants are lost to follow-up and interrupt neonatal and infant care [13].

Preparation for hospital discharge is complex and multidimensional, involving aspects of knowledge, skills, safety, and confidence in caring for children at home and navigating the service network, with a view to health outcomes and well-being of premature children and their families [14,15]. Studies indicate that it is vital to expand nurses’ confidence and knowledge to increase access to and follow-up of premature children, ensuring quality trajectories of child growth and development and adequate parental support [16].

In the Brazilian context, nurses play a central role in coordinating perinatal care and home follow-up, promoting child care, parental guidance, and family integration into the health network [17,18]. This role becomes even more challenging in small and border municipalities, where resource limitations and access difficulties may compromise adequate follow-up of premature children [3,19].

In addition to structural aspects, it is relevant to consider social influences that impact adherence to clinical follow-up, such as socioeconomic inequalities that affect attendance at neonatal follow-up appointments, highlighting the need for inclusive public policies that guarantee the right to health for vulnerable populations [2,13,20]. Recently, technological advances, such as *eHealth* tools, have been explored to strengthen parental care and facilitate remote monitoring of these newborns, which may represent a promising strategy for regions with physical and logistical barriers [21].

Given the importance of PHC as a gateway and fundamental element for continuity of care for premature infants, the need to evaluate the perceived quality of follow-up offered is emphasized, considering the perspective of professionals who work directly in these services. Tools such as the “Qualipreterm” instrument were developed and validated to instrument this evaluation and identify critical points in care [9,22]. The Qualipreterm instrument was initially proposed by Silva and Mello [9] and subsequently submitted to content validation by Paião et al. [22], with the validated version being used in the present study. Unlike previous studies, which dealt with the development and validation of the instrument, this study presents its large-scale application, with nurses from all health macro-regions of Paraná state.

In this context, Paraná state, composed of diversified municipalities and characterized by the presence of international border areas, presents a relevant scenario to investigate the perceived quality of follow-up care for premature children in PHC. Thus, the objective of the study is to analyze the perceived quality of follow-up care for premature children in PHC, from the perspective of nurses working in Paraná state, Brazil.

## Methods

### Study design

This was a cross-sectional, descriptive, and analytical study with a quantitative approach and description of spatial distribution, based on the perceived quality of follow-up care for premature children from the perspective of nurses working in PHC in Paraná state, Brazil.

### Study setting

**Fig 1:**
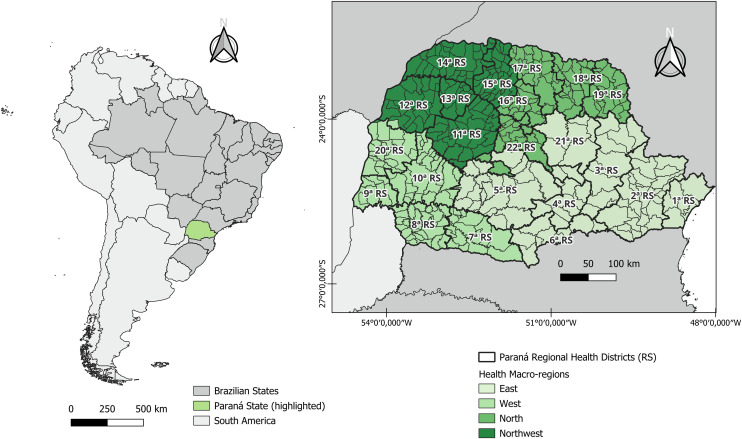
Geographic location of the state and subdivision of Health Macro-regions and Regional Health Districts. Source: Created by authors using QGIS software and public domain IBGE shapefiles.

The study was conducted in Paraná state, located in southern Brazil (Fig 1), with a territorial extension of 199,298.98 km^2^, comprising 399 municipalities and an estimated population of 11,444,380 inhabitants, resulting in a demographic density of 57.42 inhabitants per km^2^. Regarding socioeconomic indicators, the state has a Human Development Index (HDI) of 0.769, a Gini Index of 0.482, and a per capita household income of USD 348.00. Regarding basic sanitation services, 75.5% of the population has access to sewage systems, and 95.7% have access to drinking water [23].

Regarding the maternal-infant profile, Paraná state registered 139,836 live births in 2023, of which 16,137 were premature, corresponding to a prematurity rate of 11.5%. The infant mortality rate in the state was 10.7 per 1,000 live births, the third lowest in the country, below the national average of 12.4 per 1,000 live births [24].

The western portion of the state is characterized by the presence of 139 municipalities located in the border zone and border line, which corresponds to approximately 35% of the total municipalities in Paraná. This region establishes geopolitical boundaries with two countries: Paraguay to the west and Argentina to the southwest [25].

Within the organization of health services, the state is divided into four Health Macro-regions: East, West, North, and Northwest (Fig 1). In addition to the macro-regions, the state is divided into 22 Regional Health Districts, responsible for decentralized management of actions and services in the territory [25,26]. The Family Health Strategy (FHS), the main model of Primary Health Care organization in Brazil, operates through multidisciplinary teams based in Basic Health Units (BHU) and Family Health Units (FHU). In 2025, Primary Health Care coverage in the state reached 95.26%, with 2,909 Family Health Strategy teams (FHSt), 267 Primary Care teams with 20 weekly hours, and 229 teams with 30 weekly hours [27].

### Population and sample

The target population comprised nurses working in PHC in the aforementioned Brazilian state. The unit of analysis was the individual nurse, not the health service. Considering that each PHC health unit has at least one nurse in its composition, invitations were sent to a single nurse per unit to participate in the study, thus ensuring only one response per health unit and avoiding duplicate responses. The inclusion criteria were: nurses working in PHC units for at least one year. Nurses on sick leave, vacation, or leave of absence during the data collection period were excluded. In cases where more than one nurse worked in the same unit, the participant was defined by indication from the municipal manager or, in the absence thereof, by the nurse with the longest time working in the unit.

The sample size was calculated considering the total universe of 2,728 PHC units distributed in the studied setting. To determine a representative sample, a minimum of 337 participating units was established. The calculation was performed using the formula for sampling in finite populations:


$$\begin{array}{c} \text{n}=(\text{EDFF}*\text{Np}(\text{1}-\text{p})/[({\text{d}}^{\text{2}}/{\text{Z}}^{\text{2}}\text{1}-\alpha /\text{2}*(\text{N}-\text{1})+\text{p}*(\text{1}-\text{p})] \end{array}$$

Where “N” is the population size, “p” is the hypothetical frequency in % of the outcome factor in the population, “d” is the confidence limit, and “EDFF” is the design effect [28]. The statistical parameters adopted were a significance level (α) of 5%, a confidence interval of 95%, and a margin of error of 5%.

### Data collection

Data collection was conducted between February 2024 and March 2025, carried out by two researchers. Invitations to participate in the research were sent through multiple strategies: email, instant messaging (WhatsApp®), social networks, audio/video calls, through municipal managers, and in-person contact at health units. Recruitment was conducted actively and continuously throughout the entire collection period, through multiple contact strategies. Despite efforts to increase participation, not all municipalities responded, which is recognized as a study limitation and discussed in the corresponding section.

Participants responded to the questionnaire through the Google Forms® platform, structured in electronic format to facilitate access and standardize collection. The first page of the instrument presented the Free and Informed Consent Form (FICF), and by selecting “I agree”, participants automatically accessed the next page containing questions on personal and professional characterization, including: sex, age group, time since graduation, professional qualification (postgraduate degree in PHC/Family Health), length of experience in PHC, time in current unit, unit location (urban/rural), type of health unit, and frequency of care for premature children.

To evaluate the perceived quality of follow-up care for premature infants from the perspective of nurses, the “Qualipreterm” instrument was used – a guide for evaluating the quality of follow-up care for premature newborns in PHC [22]. The instrument was developed by Silva and Mello [9] and validated for content by Paião [29], with results subsequently published by Paião et al. [22], with the final validated version being used in this study. The internal consistency analysis of Qualipreterm showed an overall Cronbach's alpha of 0.989 and 0.993 for clarity and comprehensibility. The validation dissertation was completed in February 2024, prior to the beginning of data collection for this study, which occurred between March 2024 and March 2025 under approval from the Research Ethics Committee, opinion no. 6.545.705.

The instrument is structured into five fundamental domains: Domain I – Hospital discharge planning and care plan organization: Evaluates aspects related to planning the hospital-to-home transition, including family preparation, coordination between levels of care, and organization of the therapeutic plan (Cronbach's alpha 0.970–0.961); Domain II – Home follow-up through visits and telehealth: Includes home follow-up strategies, including in-person visits and telehealth resources for continuous monitoring (0.957–0.977); Domain III – Child health follow-up for health promotion and complication prevention: Covers health promotion actions, disease prevention, immunization, and early detection of complications (0.958–0.876); Domain IV – Integration between health services, education, and specialized follow-up: Analyzes intersectoral articulation and care coordination between different services and levels of care (0.972–0.984); Domain V – Family support and assistance: Evaluates support offered to families, including guidance, psychosocial support, and strengthening the family-health service bond (0.965–0.981).

Each domain is classified into four quality levels: Excellent, Good, Regular, and Inadequate, according to specific criteria established by the validated instrument.

### Data analysis

Data collected were organized in a Microsoft Excel® spreadsheet and subsequently analyzed using Jamovi software (version 2.3.28). Descriptive analyses were performed with calculation of absolute and relative frequencies for categorical variables. Statistical analysis included Pearson's chi-square tests to verify associations between the Qualipreterm domains and health macro-regions, with a significance level set at p < 0.05.

Chi-square test assumptions were verified, ensuring expected cell frequencies exceeded 5 in all analyses. Given the exploratory nature of this study, no adjustment for multiple comparisons was applied. Effect sizes were interpreted considering both statistical significance and practical relevance for health services.

The choice for bivariate analysis was based on three methodological aspects: (1) the primary objective was to describe the distribution of perceived quality of follow-up between macro-regions, not to identify predictors or causal factors; (2) the exploratory nature of the first large-scale application of the Qualipreterm instrument justifies an initial conservative approach, prioritizing characterization of patterns before complex modeling; (3) the unit of analysis was nurses’ perceptions, not the services, limiting adequate control of contextual variables that could act as confounders in multivariate models.

To describe the spatial distribution of perceived quality of follow-up, thematic maps were created using QGIS software (version 3.30.3), representing the distribution of Qualipreterm scores by health macro-region. Maps were constructed using official cartographic bases from the Brazilian Institute of Geography and Statistics (IBGE), with SIRGAS 2000 cartographic projection. Data classification was performed through proportional symbology by categories, using a differentiated color palette for each quality level (Excellent, Good, Regular, Inadequate) in each evaluated domain. This spatial representation has a descriptive character, not inferential.

### Ethical aspects

This study was conducted in accordance with the ethical principles of the Declaration of Helsinki and with the guidelines of Resolution No. 466/2012 of the National Health Council of Brazil. The project was submitted to and approved by the Research Ethics Committee, through opinion no. 6.545.705.

Data collected were stored securely and used exclusively for scientific research purposes, with access restricted to the researchers responsible for the study.

## Results

A total of 463 nurses working in PHC in Paraná state participated in the study, distributed among the four health macro-regions. The West macro-region had the highest number of participants (230; 49.7%), followed by East (118; 25.5%), Northwest (80; 17.3%), and North (35; 7.6%). The sociodemographic and professional characteristics of participants are presented in Table 1.

**Table 1:** Sociodemographic and professional characteristics of participants by health macro-region (n = 463).

Characteristic	Total n (%)	East n (%)	Northwest n (%)	North n (%)	West n (%)
**Macro-region**		118 (25.5)	80 (17.3)	35 (7.6)	230 (49.7)
**Sex**					
Female	437 (94.4)	109 (92.4)	77 (96.2)	32 (91.4)	219 (95.2)
Male	26 (5.6)	9 (7.6)	3 (3.8)	3 (8.6)	11 (4.8)
**Age group**					
Between 20 and 30 years	60 (13.0)	9 (7.6)	8 (10.0)	5 (14.3)	38 (16.5)
Between 30 and 40 years	190 (41.0)	49 (41.5)	38 (47.5)	11 (31.4)	92 (40.0)
Between 40 and 50 years	171 (36.9)	44 (37.3)	30 (37.5)	16 (45.7)	81 (35.2)
Over 50 years	42 (9.1)	16 (13.6)	4 (5.0)	3 (8.6)	19 (8.3)
**Time since graduation**					
Less than 5 years	52 (11.2)	15 (12.7)	10 (12.5)	3 (8.6)	24 (10.4)
Between 5 and 10 years	86 (18.6)	16 (13.6)	10 (12.5)	4 (11.4)	56 (24.3)
More than 10 years	325 (70.2)	87 (73.7)	60 (75.0)	28 (80.0)	150 (65.2)
**Postgraduate degree in PHC/FH**					
Yes	221 (47.7)	47 (39.8)	38 (47.5)	21 (60.0)	115 (50.0)
No	242 (52.3)	71 (60.2)	42 (52.5)	14 (40.0)	115 (50.0)
**Time working in PHC**					
Less than 5 years	161 (34.8)	32 (27.1)	26 (32.5)	11 (31.4)	92 (40.0)
Between 5 and 10 years	100 (21.6)	30 (25.4)	14 (17.5)	3 (8.6)	53 (23.0)
More than 10 years	202 (43.6)	56 (47.5)	40 (50.0)	21 (60.0)	85 (37.0)
**Time in current unit**					
Less than 5 years	295 (63.7)	69 (58.5)	44 (55.0)	18 (51.4)	164 (71.3)
Between 5 and 10 years	94 (20.3)	29 (24.6)	19 (23.8)	9 (25.7)	37 (16.1)
More than 10 years	74 (16.0)	20 (16.9)	17 (21.2)	8 (22.9)	29 (12.6)
**Unit location**					
Urban	398 (86.0)	100 (84.7)	69 (86.2)	30 (85.7)	199 (86.5)
Rural	65 (14.0)	18 (15.3)	11 (13.8)	5 (14.3)	31 (13.5)
**Type of unit**					
Family Health Strategy	340 (73.4)	85 (72.0)	57 (71.2)	27 (77.1)	171 (74.3)
Basic	107 (23.1)	32 (27.1)	20 (25.0)	8 (22.9)	47 (20.4)
Extended Basic	16 (3.5)	1 (0.8)	3 (3.8)	0 (0.0)	12 (5.2)

PHC: Primary Health Care; FH: Family Health.

From the perspective of participating nurses, the evaluation of perceived quality of follow-up care for premature children in PHC showed important variations among the five evaluated domains (Table 2). Domain I (hospital discharge planning and care plan organization) presented the most concerning performance, with 273 (58.9%) evaluations classified as inadequate and only 43 (9.3%) achieving good quality standards. Domain II (home follow-up through visits and telehealth) demonstrated predominantly regular performance, with 277 (59.8%) evaluations in this category, 135 (29.2%) classified as good, and 50 (10.8%) as inadequate.

**Table 2:** Qualipreterm scores across the five domains in Paraná state, Brazil, 2025.

Domain	Inadequate n (%)	Regular n (%)	Good n (%)	Excellent n (%)
I. Hospital discharge planning and care plan organization	273 (58.9%)	147 (31.7%)	43 (9.3%)	0 (0%)
II. Home follow-up through visits and telehealth	50 (10.8%)	277 (59.8%)	135 (29.2%)	1 (0.2%)
III. Child health follow-up for health promotion and complication prevention	8 (1.7%)	261 (56.4%)	193 (41.7%)	1 (0.2%)
IV. Integration between health services, education, and specialized follow-up	65 (14.0%)	277 (59.8%)	120 (25.9%)	1 (0.2%)
V. Family support and assistance	37 (8.0%)	139 (30.0%)	271 (58.5%)	16 (3.5%)

Domain III (child health follow-up for health promotion and complication prevention) exhibited better performance, with 193 (41.7%) evaluations classified as good, 261 (56.4%) as regular, and only 8 (1.7%) as inadequate. Domain IV (integration between health services, education, and specialized follow-up) showed intermediate results, with 120 (25.9%) good evaluations, 277 (59.8%) regular, and 65 (14.0%) inadequate. Domain V (family support and assistance) demonstrated the best overall performance, with 271 (58.5%) evaluations classified as good, 139 (30.0%) regular, 37 (8.0%) inadequate, and notably achieved the highest proportion of excellent classifications with 16 (3.5%).

Analysis by health macro-region revealed consistent weaknesses in Domain I across all regions (Table 3). The West macro-region presented the most critical scenario, from the nurses’ perspective, with 63.0% of evaluations as inadequate, followed by the East macro-region (62.5%). The North macro-region recorded 47.5% of evaluations classified as inadequate, while the Northwest presented 45.7%. Regarding evaluations classified as good, the Northwest macro-region obtained the best performance (15.0%), followed by West (10.0%), North (5.7%), and East (5.1%). No macro-region achieved excellent classification in this domain.

**Table 3:** Analysis of association between health macro-region and quality of follow-up care domains for premature infants in Primary Health Care, Paraná, Brazil, 2024-2025 (n = 463).

Domain	χ²	Df	p-value
Domain I: Hospital discharge planning	15.0	6	0.020*
Domain II: Home follow-up	24.0	9	0.004*
Domain III: Child health follow-up	17.7	9	0.039*
Domain IV: Integration between services	19.2	9	0.023*
Domain V: Family support and assistance	13.2	9	0.154

Pearson's chi-square test. χ² = chi-square; df = degrees of freedom. * p < 0.05 (statistically significant).

**Fig 2:**
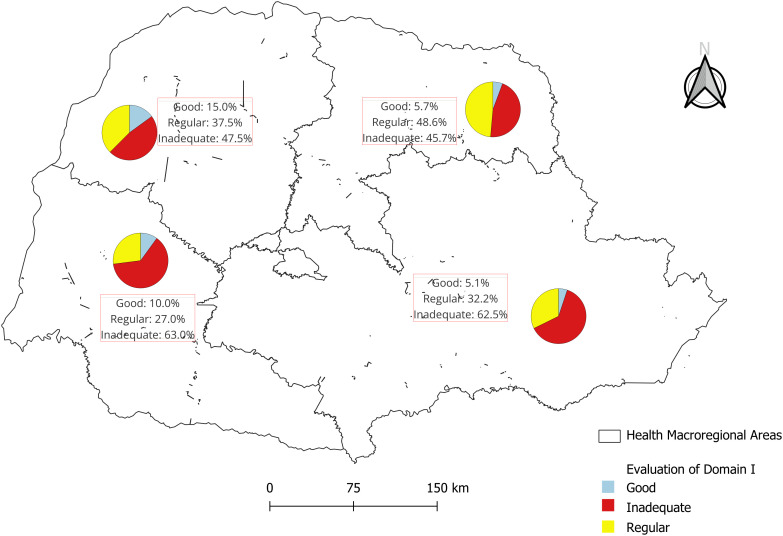
Distribution of Domain I scores according to health macro-regions of Paraná, Brazil, 2025. Source: Created by authors using QGIS software and public domain IBGE shapefiles.

**Fig 3:**
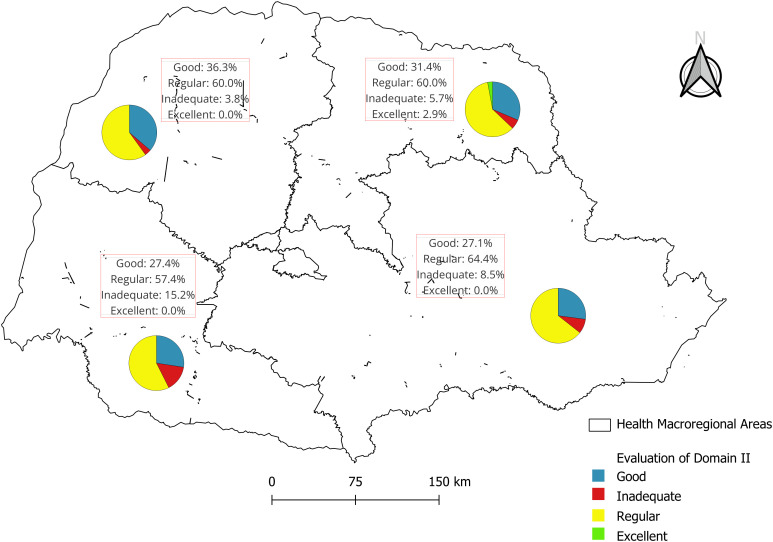
Distribution of Domain II scores according to health macro-regions of Paraná, Brazil, 2025. Source: Created by authors using QGIS software and public domain IBGE shapefiles.

**Fig 4:**
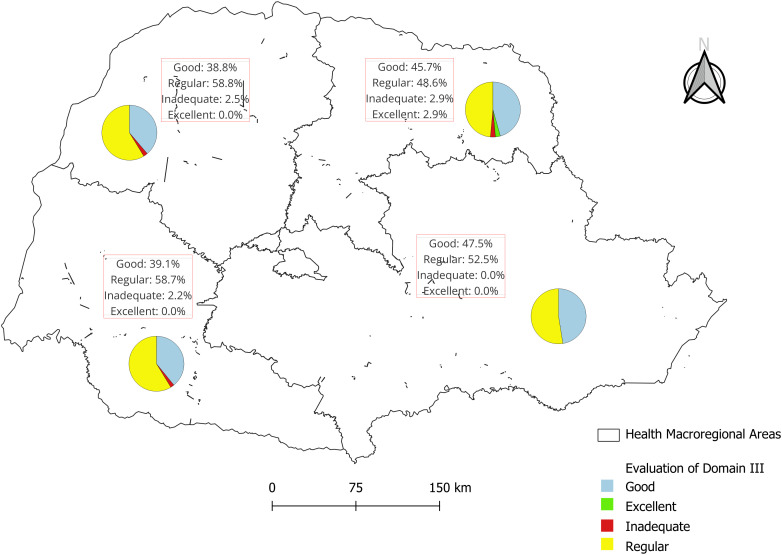
Distribution of Domain III scores according to health macro-regions of Paraná, Brazil, 2025. Source: Created by authors using QGIS software and public domain IBGE shapefiles.

**Fig 5:**
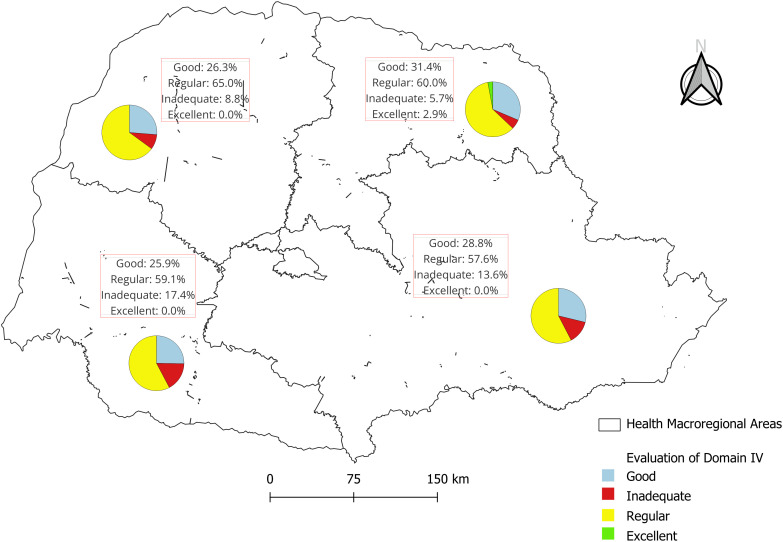
Distribution of Domain IV scores according to health macro-regions of Paraná, Brazil, 2025. Source: Created by authors using QGIS software and public domain IBGE shapefiles.

**Fig 6:**
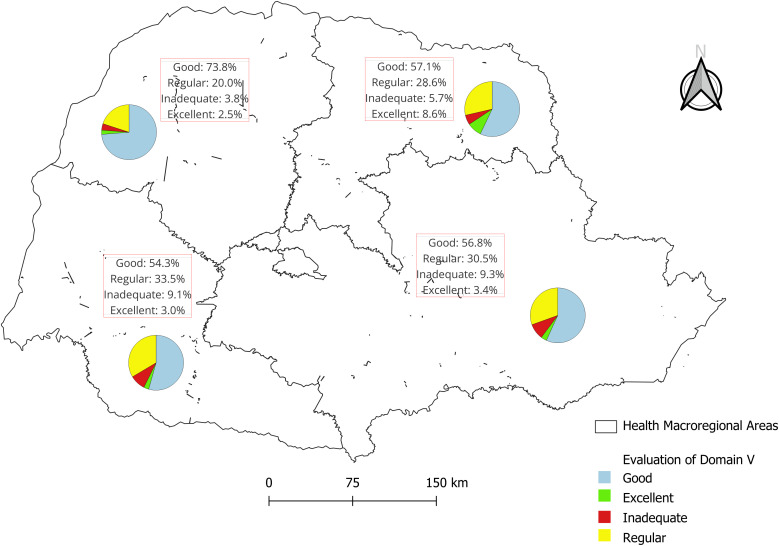
Distribution of Domain V scores according to health macro-regions of Paraná, Brazil, 2025. Source: Created by authors using QGIS software and public domain IBGE shapefiles.

The association analysis between health macro-region and perceived quality of domains revealed statistically significant differences in four of the five evaluated domains (Table 3). Domain II (Home Follow-up) presented the strongest association with macro-region (χ² = 24.0; p = 0.004), followed by Domains IV (Service Integration: p = 0.023), III (Child Health Follow-up: p = 0.039), and I (Hospital Discharge Planning: p = 0.020). Domain V (Family Support and Assistance) showed no significant variation among macro-regions (p = 0.154). The spatial representation of these differences is presented in Figs 2–6.

Domain II (home follow-up through visits and telehealth) presented a more balanced distribution among categories (Fig 3). The Northwest macro-region demonstrated the best performance with 36.3% of evaluations evaluated as good and only 3.8% inadequate. The North macro-region achieved 31.4% of evaluations with good evaluation and 5.7% inadequate, while the East obtained 27.1% of evaluations considered good and 8.5% inadequate. The West macro-region, despite 27.4% of evaluations evaluated as good, recorded the highest percentage of inadequacy (15.2%). The regular category predominated in all regions (57.4%−64.4%).

Domain III showed substantial improvement in performance (Fig 4). The East macro-region stood out with 47.5% of evaluations classified as good with no inadequate evaluations. The North macro-region achieved 45.7% of evaluations considered good with only 2.9% inadequate, followed by West (39.1% good; 2.2% inadequate) and Northwest (38.8% good; 2.5% inadequate). The North and East macro-regions achieved excellent classification (2.9% each).

Domain IV revealed moderate performance with regional variations (Fig 5). The North macro-region maintained leadership with 31.4% of evaluations classified as good and achieved 2.9% excellent classification. The East macro-region presented 28.8% of evaluations as good but recorded the highest percentage of inadequacy (13.6%). The Northwest (26.3% good; 8.8% inadequate) and West (25.9% good; 17.4% inadequate) macro-regions completed the scenario. The regular category predominated (57.6%−65.0%).

Domain V presented the best overall performance (Fig 6). The Northwest macro-region achieved exceptional results with 73.8% of evaluations considered good and only 3.8% inadequate. The North macro-region maintained 57.1% of evaluations as good, 5.7% inadequate, and the highest percentage of excellent classification (8.6%). The East (56.8% good; 9.3% inadequate) and West (54.3% good; 9.1% inadequate) macro-regions showed similar performances. This domain was the only one to present excellent classification in three macro-regions (2.5%−8.6%).

The cross-sectional analysis revealed distinct patterns of regional performance from the nurses’ perspective. The North macro-region demonstrated greater consistency in quality, standing out in Domains III, IV, and V. The Northwest macro-region, despite difficulties in Domain I, presented excellence in Domain V and good performance in Domain II. The East macro-region showed polarization, with excellent performance in Domain III but weaknesses in Domain IV. The West macro-region presented more homogeneous performance, but with greater challenges in Domain I.

## Discussion

The evaluation of perceived quality of follow-up care for premature children in PHC in the studied setting, from the perspective of nurses, demonstrated significant heterogeneity among the domains evaluated by the Qualipreterm instrument. The results revealed critical weaknesses in care planning (Domain I) and intermediate performance in home follow-up (Domain II), while family support (Domain V) stood out positively. These findings are consistent with Brazilian studies that identified gaps in continuity of care for premature infants, especially in the transition to service points in the health network [18,19,30].

The inadequate performance in Domain I reflects global challenges in the hospital-to-home transition for premature infants. Studies confirm that hospital discharge planning remains a critical weakness in health systems, identifying that discharge criteria and practices vary significantly between and within neonatal units, constituting a predominantly professional transitional process with low family participation [31]. This finding corroborates the findings of the present study, with 58.9% of evaluations classified as inadequate in this domain.

Changes in care assessment and satisfaction with discharge preparation in neonatal units are critical for effective follow-up [32]. On the other hand, analysis of the variety of follow-up programs for very preterm children evidenced the need for standardized protocols that seek to ensure better quality of neonatal and infant care [4]. In the present study, the perceived inadequacy in discharge planning observed in all macro-regions suggests the implementation of structured protocols, aligned with recommendations on adequate preparation for discharge of vulnerable children [33].

Well-planned hospital discharge for premature children represents a fundamental element for ensuring effective continuity of care in PHC. Adequate planning, initiated early during hospitalization, empowers families with the knowledge and skills necessary for home care, reduces parental anxiety, and strengthens confidence in managing the child's specific needs [32,33].

Successful transition from the hospital environment to home not only contributes to better health outcomes, considering that premature children are at increased risk for neurosensory deficiencies and delays in motor, language, and social development [5], but also empowers families to exercise competent and safe parenting [15,34]. Thus, the process transforms a potentially traumatic experience into an opportunity to strengthen family bonds and promote the child's comprehensive health.

The differences found among the studied macro-regions reflect structural and organizational inequalities in health services as perceived by nurses. The North macro-region presented more consistent performance, particularly in Domains III and V, while the West macro-region faced greater challenges in Domain I. These disparities are corroborated by studies demonstrating significant regional variations in the quality of maternal and child care in Brazil [35,36]. Border regions face specific vulnerabilities related to intense population movement and cultural diversity, factors that can impact continuity of care [3].

Analysis of the macro-regions revealed that border contexts present unique challenges for follow-up of premature infants. Discontinuity in care for premature children occurs in border regions, related to the complexities of cross-border health systems [12]. In the present study, the perceived disparities found among the analyzed macro-regions reflect not only organizational differences but also specific sociodemographic and economic characteristics of each territory.

The better perceived performance in Domain V (family support) aligns with evidence on the importance of family support in the care of premature infants. Positive experiences of mothers and health professionals in the third stage of the kangaroo method were related to structured family support [18]. Interventions focused on family support resulted in better outcomes for premature children [37–39], including reduction of parental stress and improvement in care efficacy.

The findings of the present study converge with recent international recommendations for the care of premature children, with interventions related to the kangaroo method immediately after birth, early initiation of breastfeeding, and fundamental family involvement, which can substantially reduce mortality in premature and low birth weight infants. WHO recommendations emphasize that mothers and newborns should remain together from birth, not being separated unless the baby is critically ill, and highlight the need for improvements in family support, including education, counseling, peer support, and home visits by trained professionals [40]. Furthermore, the FIGO PremPrep-5 initiative is emphasized as an effective and relatively simple intervention to improve outcomes for premature infants in various contexts, particularly in low- and middle-income countries where more than 80% of preterm births occur [41]. This international evidence supports the importance of strengthening Domain V in the present study, suggesting the need for increased opportunities for expansion of evidence-based practices in the Brazilian context.

The intermediate performance of Domain II expresses both the potentialities and challenges perceived by nurses in implementing innovative home follow-up strategies. The COVID-19 pandemic globally accelerated the adoption of telehealth in neonatal care, demonstrating its viability as a complementary tool to in-person follow-up [5,42,43]. Studies identified that telemedicine interventions, with real-time audiovisual communication and eHealth applications, have the potential to improve parental well-being by increasing self-efficacy and discharge readiness, in addition to reducing anxiety and stress [43]. Management systems based on WeChat mini-programs were applied for follow-up of catch-up growth recovery in premature infants after discharge [44]. Video consultations were identified as a viable means of providing early discharge programs for premature infants [45]. In the international context, an Israeli study demonstrated promising potential for using WhatsApp® in post-discharge follow-up of premature babies [46]. The incorporation of these digital technologies can substantially strengthen home follow-up and improve its quality, especially in regions with geographic barriers such as the border areas studied here.

The regular performance in Domain IV (integration between services) evidences perceived weaknesses in care coordination between different points of care. Gaps in the referral and counter-referral system can compromise the comprehensiveness of care in the health care network [47]. Studies indicate that an effective referral system ensures a close relationship between all levels of care, and individuals have the possibility of receiving the best care [48].

The perceived weaknesses identified in Domain IV reflect a global problem in care coordination for premature children. Insufficient continuity of care for premature infants discharged from neonatal units includes challenges in coordination between hospital services and PHC and dependence of families on hospital outpatient follow-up, to the detriment of expanded primary health care follow-up [30]. The family preference for hospital follow-up, also observed in the present study, results in care discontinuity and care fragmentation. Specific monitoring of premature children is important, emphasizing that PHC professionals play a relevant role in longitudinal, coordinated, and timely care during early childhood, including assessment of growth, development, feeding and behavior, mitigation of functional limitations, and determination of appropriate specialized supports [49]. Implementation of structured frameworks such as this can strengthen integration between services, given the findings in the analyzed context.

Integration between health and education, an important component of Domain IV, proved particularly challenging. Implementation of early development interventions after hospital discharge to prevent motor and cognitive deficiencies in premature infants requires a multidisciplinary approach and coordination between different sectors [50]. Collaborative partnerships between the health sector and early intervention programs are essential to optimize developmental outcomes in at-risk children, requiring co-management and joint monitoring of services provided [51]. Care coordination and community services based on early intervention, in addition to supports for caregivers and families, can mitigate postnatal psychosocial complications associated with developmental sequelae [49]. In the present study, weakness in this domain indicates the need to strengthen intersectoral partnerships.

The findings suggest the need for differentiated investments by domain and region. Strengthening hospital discharge planning requires standardized protocols, professional training, and better coordination between service network points and other institutions, with systematic interventions being necessary [30].

The regional variability identified suggests that public policies should consider territorial specificities. The importance of understanding the right to health will bring contributions to border regions, with the development of more effective policies [52]. Specific complexities of international border regions, including migratory aspects and cultural diversity, impact the organization of different services [19].

Despite the Paraná Maternal and Child Care Line, formerly called the Paraná Mother Network Program (PRMP), having from its conception envisioned the qualification of professionals through continuing education programs [53], evaluation studies of program implementation identified important weaknesses related to professional qualification [54,55]. Frank et al. [54] demonstrated that program implementation remains incipient, especially regarding professional qualification. No studies were identified in the literature that specifically evaluate training programs for PHC nurses on follow-up of premature infants in Paraná state, evidencing an important knowledge gap in this area. This absence of structured continuing education programs specific to follow-up of premature infants may contribute to explaining the observed heterogeneity in perceived quality of care among the different macro-regions of the state.

The 17% response rate, although resulting in a sample 37% higher than the required statistical minimum (n = 463 versus n = 337), may introduce selection bias. Despite efforts to increase participation through multiple recruitment strategies, not all municipalities responded to the invitation, which may mean that participating nurses have different characteristics from those who did not respond, potentially overestimating or underestimating follow-up quality in certain regions.

The cross-sectional design limits the ability to establish causal relationships between studied variables and prevents evaluation of temporal changes in follow-up quality. Longitudinal studies would be necessary to evaluate the impact of interventions and the evolution of quality over time. Additionally, the specific geographic focus on Paraná state limits generalization of findings to other Brazilian or international contexts, especially considering regional specificities, such as the presence of border areas that may not be representative of other realities.

The unit of analysis was nurses’ individual perceptions of follow-up quality, not the health services or their objective organizational characteristics. This methodological choice, although aligned with the objective of capturing the professional perspective on care quality, limits the ability to adequately control contextual variables of services (infrastructure, resources, organizational model) that could act as confounders in multivariate models, justifying the choice for bivariate analysis. Future studies with specific designs aimed at identifying predictors can explore causal relationships using multivariate approaches, having the descriptive findings of this study as empirical basis.

Additionally, the spatial representation of presented data has a descriptive character, not constituting inferential spatial analysis with application of spatial statistical methods. Thematic maps allow visualization of geographic distribution patterns of perceived quality but do not enable inferences about spatial autocorrelation or neighborhood effects.

The study also did not include the perspective of families nor objective evaluations of clinical outcomes of premature infants, which could provide a more comprehensive view of follow-up quality and its real effectiveness.

Despite these limitations, this study contributes significantly to Brazilian literature by providing systematic evaluation of perceived quality of follow-up for premature infants using a validated instrument [9,22,29]. The state-wide scope and analysis by macro-regions allow identification of regional patterns and inputs for differentiated policies. Use of Qualiprematuro enables future comparisons and monitoring of service improvements.

Future perspectives for improvement of follow-up of premature infants include implementation of integrated care models. Bourque et al. [56] analyzed best practices for discharge of high-risk infants in regional neonatal intensive care units, identifying key elements for safe transition. International experience with structured post-discharge follow-up programs, including responsive parenting programs [52,53] and multidisciplinary follow-up interventions [57–59], offers models adaptable to the Brazilian context. Moen [60] questions how to achieve safe discharge of premature infants without unnecessary delays, emphasizing the need for balance between safety and discharge opportunity. Implementation of validated tools such as Qualipreterm, used in the present study, enables systematic monitoring of service quality and identification of priority areas for intervention. The development of public policies based on scientific evidence can contribute to ensuring adequate follow-up of premature infants throughout Paraná territory. Such policies should consider regional specificities and socioeconomic inequalities identified in this study.

Development of specific policies for border regions should consider binational agreements, standardization of cross-border protocols, and strategies for overcoming linguistic and cultural barriers [61–63]. Expansion of FHS coverage in large municipalities could reduce identified disparities, taking advantage of advantages observed in smaller municipalities.

## Conclusions

This study identified significant heterogeneity in the perceived quality of follow-up care for premature children in the PHC network in the studied context, from the perspective of nurses. Care planning emerged as the most critical domain, with the majority of evaluations classified as inadequate in all analyzed macro-regions, indicating perceived systemic weakness in the hospital-to-home transition. In contrast, family support and assistance demonstrated better performance, evidencing potential for strengthening this dimension of care, from the perspective of the participating nurses.

The identified regional disparities reflected structural and organizational inequalities in health systems, as perceived by nurses, with differences between greater consistency in quality and care challenges. These variations indicate the need for differentiated public policies that consider territorial specificities, including characteristics of border areas that present specific vulnerabilities related to population movement and cultural diversity.

The findings suggest the need for implementation of standardized protocols for expanded care planning, strengthening coordination between different points of care in the service network, and enhancement of innovative strategies such as telehealth to support home follow-up. Use of the Qualiprematuro instrument proved useful for systematic evaluation of perceived quality of follow-up, enabling identification of priority areas for intervention and longitudinal monitoring of improvements in care for premature children and their families.

The perspective of PHC nurses provided important information on weaknesses and potentialities of the system, highlighting the central role of these professionals in coordinating care for premature children and their families. Investments in continuing health education, clinical decision support tools, and strengthening support networks are essential to qualify follow-up and ensure better health outcomes for this vulnerable population.
