# Supplementary material for: Quality of follow-up care for premature infants in primary health care: A cross-sectional study from nurses' perspective in Paraná state, Brazil
Source: PLoS One. 2026 Jun 10;21(6):e0351395. doi: 10.1371/journal.pone.0351395 (PMC13252751; doi:10.1371/journal.pone.0351395)
Supplement: Attachment — Submitted filename: Response_Letter.docx [file pone.0351395.s002.docx]

Dear Editors,

We sincerely thank you and the three reviewers for the thorough and constructive evaluation of our manuscript entitled “Quality of follow-up care for premature infants in primary health care: A cross-sectional study from nurses’ perspective in Paraná state, Brazil” (PONE-D-25-67906).

We greatly appreciate the detailed feedback, which has been instrumental in strengthening the manuscript. We have carefully addressed all comments and recommendations from the editor and reviewers. Below, we provide a detailed point-by-point response, indicating how each comment was addressed and specifying the exact location of corresponding modifications in the revised manuscript.

We believe these revisions have substantially improved the quality and clarity of our work, and we hope the manuscript is now suitable for publication in PLOS ONE.

RESPONSE TO EDITORIAL REQUIREMENTS

1. Data Availability

Editorial requirement: “Compliance with PLOS ONE’s Data Availability Policy is mandatory. Authors must deposit the anonymized dataset and relevant materials in a public repository.”

Response: We have fully complied with PLOS ONE’s Data Availability Policy. The complete anonymized dataset has been deposited in the Open Science Framework (OSF) repository and is now publicly available at: - URL: https://osf.io/68zwh/ - DOI: 10.17605/OSF.IO/68ZWH

Dataset content: The dataset includes anonymized individual-level data on: - Nurses’ sociodemographic characteristics (age, gender, educational level, years of experience) - Professional characteristics (employment type, workload, training in premature infant care) - Assessment scores for all five domains of the Qualipreterm instrument - Classification by macro-region (West, East, Northwest, North).

Anonymization procedures: To preserve participant confidentiality in small municipalities, the following variables were excluded from the public dataset: - Municipality name - Health unit name - Specific geographic coordinates.

This anonymization was required by the Research Ethics Committee (opinion number 6.545.705) to protect the identity of participants in small municipalities where individual nurses could potentially be identified.

Changes in manuscript: - Data Availability Statement section added after Conclusions.

2. Funding Information

Editorial requirement: “Please ensure that funding information is not included in Acknowledgments or elsewhere in the manuscript.”

Response: We have removed all funding-related text from the Acknowledgments section. Funding information remains only in the Editorial Manager system, as required.

Revised Acknowledgments: The Acknowledgments section now states: “We thank the nurses who participated in this study for their valuable contributions and the Municipal Health Departments of Paraná state for their support in data collection.”

Funding Statement (for Editorial Manager system only): “This study was funded by the Coordination for the Improvement of Higher Education Personnel - Brazil (CAPES) - Funding Code 001, through a postdoctoral fellowship granted to the first author. The funders had no role in study design, data collection and analysis, decision to publish, or preparation of the manuscript.”

Changes in manuscript: - Acknowledgments section revised (page 28, lines 632).

3. Copyright Compliance for Maps

Editorial requirement: “We note that Figures 1-6 in your submission contain map images that may be copyrighted.”

Response: All maps (Figures 1-6) were created by the authors using: - QGIS software (open-source, version 3.30.3) - Public domain shapefiles from IBGE (Brazilian Institute of Geography and Statistics)

No proprietary or copyrighted map data (such as Google Maps) was used. All spatial data are from public domain sources. The maps are original creations by the authors and can be published under CC BY 4.0 license without restrictions.

Changes in manuscript: - Figure captions updated to include source information: “Created by authors using QGIS software and public domain IBGE shapefiles” (Figures 1-6, pages 7, 16-18).

RESPONSE TO CONCEPTUAL AND METHODOLOGICAL REQUIREMENTS

4. Conceptual Clarification: Perceived vs. Objective Quality

Editorial requirement: “The manuscript should clearly state throughout that the study evaluates perceived quality of follow-up care from nurses’ perspective, not objective service quality.”

Response: We have revised the entire manuscript to consistently emphasize that this study evaluates nurses’ perceptions of quality, not objective measurements of service quality.

5. Unit of Analysis and Sampling Procedures

Editorial requirement: “The manuscript should explicitly clarify that the unit of analysis is the individual responding nurse, not the health service.”

Response: We added a dedicated paragraph in the Methods section clearly stating the unit of analysis and describing procedures to ensure data quality.

Changes in manuscript: - Methods - Population and sample section: Added explicit statement “The unit of analysis was the individual nurse, not the health service” (page 8).

6. Internal Consistency Analysis

Editorial requirement: “Include internal consistency analysis of the Qualipreterm instrument in the current sample (e.g., Cronbach’s alpha for each domain).”

Response: We inserted Cronbach’s alpha for each of the five domains of the Qualipreterm instrument.

Changes in manuscript: - Methods - Data collection section: Added paragraph reporting Cronbach’s alpha values: overall instrument (0.989), clarity/comprehensibility (0.993), and alpha values for each domain: Domain I (0.970-0.961), Domain II (0.957-0.977), Domain III (0.958-0.876), Domain IV (0.972-0.984), Domain V (0.965-0.981) (page 10).

7. Justification for Bivariate Analysis Approach

Editorial requirement: “Authors are strongly encouraged to include additional statistical analyses, such as multivariate models. If such analyses are not conducted, a clear methodological justification should be provided.”

Response: We added a comprehensive methodological justification for our analytical approach in the Methods section.

This study primarily employed descriptive statistics and bivariate analyses (chi-square tests) rather than multivariate modeling for the following methodological reasons:

Descriptive nature of the study: The primary objective was to provide a comprehensive description of nurses' perceptions of follow-up care quality throughout Paraná state, identifying domains with greatest deficiencies and geographic variations. This descriptive objective is appropriately addressed through frequency distributions and bivariate comparisons.

Sample characteristics: Although the overall sample size is adequate (n=463), the distribution among the four macro-regions is unequal (West: 230; East: 118; Northwest: 80; North: 35). Multivariate models including multiple categorical predictors would result in small cell sizes for some region-covariate combinations, potentially producing unstable estimates.

Nature of the outcome variable: The Qualipreterm instrument produces categorical classifications (adequate/inadequate) for each domain. The high prevalence of "inadequate" classifications in some domains (>80% in Domain I) creates challenges for logistic regression modeling due to quasi-complete separation problems.

Exploratory focus: As the first large-scale application of the Qualipreterm instrument, this study aims to map the current landscape of follow-up care quality rather than test specific causal hypotheses. Multivariate models would be more appropriate in hypothesis-testing studies examining specific determinants of quality.

Changes in manuscript: - Methods - Data analysis section: Added complete justification paragraph (page 11).

8. Spatial Analysis Terminology

Editorial requirement: “Revise terminology used to describe spatial methods, replacing expressions like ‘spatial analysis’ with ‘spatial visualization’ when appropriate.”

Response: We revised all terminology related to spatial methods throughout the manuscript to accurately reflect that the maps are descriptive visualizations and not inferential spatial analyses.

Specific changes: - “spatial analysis” → “spatial visualization” - “spatial distribution analysis” → “geographic distribution mapping” - “geoprocessing analysis” → “geographic information system (GIS) mapping”

Clarification added in Methods: “Geographic Visualization Maps were created using QGIS software (version 3.30.3) and publicly available shapefiles from the Brazilian Institute of Geography and Statistics (IBGE). These maps provide descriptive geographic visualization of the distribution of quality classifications throughout Paraná state. No inferential spatial statistical methods (such as spatial autocorrelation analysis, hot-spot detection, or spatial regression) were applied. The maps serve only to illustrate the geographic pattern of nurses’ quality assessments.”

Changes in manuscript: - Methods - Data analysis section: Added clarification that “This spatial representation has a descriptive character, not inferential” (page 12). - Throughout manuscript: Replaced “spatial analysis” terminology with “spatial visualization” or “geographic distribution”.

9. Expanded Discussion of Limitations

Editorial requirement: “Expand the limitations section to explicitly address: the self-reported nature of data; potential social desirability bias; response rate and related selection bias; inherent limitations of cross-sectional design.”

Response: We substantially expanded the Limitations section in the Discussion to comprehensively address all requested issues.

Revised Limitations section: “Limitations This study has several important limitations that should be considered when interpreting the findings (pages 24-26).

RESPONSE TO REVIEWER #1

Reviewer #1 - Comment 1: Clarifying Novelty and Contribution

Reviewer comment: “The manuscript should more explicitly differentiate how this study’s application of the Qualipreterm instrument differs from previous validation studies and what new knowledge it contributes to the literature.”

Response: We added clarification distinguishing this study from previous validation work.

Text added (Introduction): “The Qualipreterm instrument was initially proposed by Silva and Mello [9] and subsequently submitted to content validation by Paião et al. [22], with the validated version being used in the present study. Unlike previous studies, which dealt with the development and validation of the instrument, this study presents its large-scale application, with nurses from all health macro-regions of Paraná state. This is the first study to apply the Qualipreterm at a state-wide scale to systematically evaluate perceived quality of follow-up care for premature infants in primary health care settings.”

Changes in manuscript: - Introduction: Added distinguishing paragraph about study novelty (page 6).

Regarding the study's contribution:

This study makes several innovative contributions to the literature on premature infant follow-up care. Although the Qualipreterm instrument has been validated, this is the first large-scale application to evaluate actual service delivery across a diverse geographic region. Previous studies focused primarily on instrument development and psychometric validation; this study translates that validated tool into actionable evidence about real-world practice.

The identification of Domain I (hospital discharge planning) as the most deficient area across all regions provides specific direction for quality improvement efforts. Similarly, the finding that Domain V (Family support and assistance) is the best-performing domain suggests that relationship-centered aspects of care are being prioritized, even in resource-limited contexts.

The observed geographic variations, particularly the poorer performance in the North macro-region, highlight the need for context-specific interventions that address regional disparities in health system capacity and professional training.

Reviewer #1 - Comment 2: Relationship with Previous Publications

Reviewer comment:

"Please clarify: Which publication reported the initial development of the Qualipreterm guide? Which publication reported the content validation? Did the version used in this study correspond to the final validated version?"

Response: We added a dedicated paragraph in the Methods section clarifying the relationship between this study and previous publications about the Qualipreterm instrument.

Text added (Methods section):

"Qualipreterm Instrument: Development and Validation History

The Qualipreterm instrument was developed and validated through a multiphase process reported in previous publications:

Initial development: The instrument was initially conceptualized based on clinical guidelines and systematic literature review.

Content validation: Content validity was established through a Delphi consensus process with expert panel evaluation, reported in [references 22, 29]. This process confirmed that the instrument adequately covers the key domains of follow-up care for premature infants in primary health care.

The version of the Qualipreterm instrument used in the current study is the final validated version resulting from the content validation process. No modifications were made to the instrument for this application study. The instrument consists of 64 items across five domains: (I) Hospital discharge planning, (II) Home follow-up care, (III) Growth and development surveillance, (IV) Family guidance, and (V) Family support and assistance.

This study represents the first large-scale application of the validated instrument to evaluate actual service delivery."

Reviewer #1 - Comment 3: Timeline and Ethical Considerations

Reviewer comment:

"The application evaluation reported in this manuscript appears to have been conducted close to or overlapping with the validation process of the assessment tool. How was the timeline of validation and application managed? Did ethical approval cover the use of the questionnaire at that stage?"

Response: We added clarification about the timeline and ethical approval coverage.

Text added:

"Timeline and Ethical Approval

Content validation of the Qualipreterm instrument was completed in February 2024. Data collection for the current application study was conducted from March 2024 to March 2025, after completion of the validation process.

The research protocol for this application study was reviewed and approved by the Research Ethics Committee of the State University of Western Paraná (opinion number 6.545.705, date: November 2023). The ethical approval explicitly covered the use of the final validated version of the Qualipreterm instrument to evaluate follow-up care quality in primary health care services throughout Paraná state.

All participants provided informed consent, which clearly stated the study purpose, the voluntary nature of participation, and procedures to ensure confidentiality. Participants were informed that the data would be used to evaluate follow-up care quality and inform health policy recommendations."

Changes in manuscript: Methods section: Timeline and Ethical Approval paragraph added (page 11).

RESPONSE TO REVIEWER #2

Reviewer #2 - Comment on Unit of Analysis

Reviewer comment: “The unit of analysis is not clear. Although nurses provided individual responses, the analyses and interpretations implicitly treat the data as representative of health services.”

Response: We explicitly clarified the unit of analysis and addressed procedures for handling potential duplicate responses from the same health unit. (See complete response to Editorial Requirement #5 above - the Methods section now includes dedicated paragraph on unit of analysis and procedures to avoid duplicates)

Reviewer #2 - Comment on Statistical Analysis

Reviewer comment: “The exclusive use of descriptive statistics and chi-square tests, without multivariate modeling, does not support the conclusions about regional differences.”

Response: We added a comprehensive methodological justification for our analytical approach. (See complete response to Editorial Requirement #7 above - the Methods section now includes detailed justification for bivariate approach)

We also revised interpretative statements to avoid overstatement of causal conclusions:

Revised original statements: - “Regional differences were caused by…” → “Regional differences were observed, which may reflect…”

Changes in manuscript: - Methods - Data analysis section: Statistical Analysis Approach section added (page 10). - Results and Discussion: Interpretative statements revised to reflect associational rather than causal language (pages 12-20).

Reviewer #2 - Comment on Spatial Analysis

Reviewer comment: “The spatial analysis is purely descriptive. No spatial statistical methods were applied.”

Response: We completely agree and revised all terminology and clarified that the maps are only descriptive visualizations. (See complete response to Editorial Requirement #8 above - terminology revised throughout; Methods section includes clarification that no inferential spatial methods were used)

RESPONSE TO REVIEWER #3

Reviewer #3 - Comment 1: Pediatric Population Data

Reviewer comment: “Authors should provide an explanation of the pediatric population, number of births, number of premature births, and premature mortality rate for the entire state as well as for each region.”

Response: We added comprehensive demographic and epidemiological data for Paraná state in the Study Setting section.

Text added: “Pediatric Population and Birth Statistics According to DATASUS (Brazilian Ministry of Health Information System) data for the year 2023:

Paraná State (overall): - Total population: approximately 11.5 million - Live births: 139,836 - Premature births (<37 weeks): 16,137 (11.5% of live births) - Infant mortality rate: 10.7 per 1,000 live births - Neonatal mortality rate: 7.2 per 1,000 live births

These data highlight the substantial burden of prematurity in Paraná state, with more than 16,000 premature infants requiring specialized follow-up care annually.”

Changes in manuscript: - Methods - Study setting section: Added comprehensive maternal-infant demographic data (page 6).

Reviewer #3 - Comment 2: Participant Characteristics Table

Reviewer comment: “Background information about the primary health care nurses who participated in the study would be better presented in a table. Additionally, the number of nurses who participated from each of the four regions should also be presented.”

Response: We created a comprehensive table presenting participant characteristics, including distribution by macro-region.

Text in Results section: “Table 1 presents the sociodemographic and professional characteristics of the 463 participating nurses. Distribution among macro-regions was unequal, with nearly half of respondents (49.7%) from the West region, while the North region contributed only 7.6% of participants.”

Changes in manuscript: - Results section: Table 1 added presenting complete participant characteristics by macro-region (page 12).

Reviewer #3 - Comment 3: Educational System for Nurses

Reviewer comment: “Is there any common educational system for primary health care nurses regarding follow-up care for premature infants at the national or state level?”

Response: We added information about professional training systems in the Discussion section.

Text added: “Despite the Paraná Maternal and Child Care Line, formerly called the Paraná Mother Network Program (PRMP), having from its conception envisioned the qualification of professionals through continuing education programs [53], evaluation studies of program implementation identified important weaknesses related to professional qualification [54,55]. Frank et al. [54] demonstrated that program implementation remains incipient, especially regarding professional qualification. No studies were identified in the literature that specifically evaluate training programs for PHC nurses on follow-up of premature infants in Paraná state, evidencing an important knowledge gap in this area. This absence of structured continuing education programs specific to follow-up of premature infants may contribute to explaining the observed heterogeneity in perceived quality of care among the different macro-regions of the state.”

Changes in manuscript: - Discussion section: Added professional training context paragraph (page 24).

We sincerely hope that these comprehensive revisions have addressed all concerns raised by the editor and reviewers. We believe the manuscript is now substantially improved and makes a valuable contribution to the literature on premature infant follow-up care in primary health care settings.

We appreciate your consideration and look forward to your response.

Sincerely,

Ivaneliza Simionato de Assis and co-authors
