# Supplementary material for: Quality of follow-up care for premature infants in primary health care: A cross-sectional study from nurses' perspective in Paraná state, Brazil
Source: PLoS One. 2026 Jun 10;21(6):e0351395. doi: 10.1371/journal.pone.0351395 (PMC13252751; doi:10.1371/journal.pone.0351395)
Supplement: Peer review history

# Peer review: Decision Letter 0

**DOI:** 10.1371/journal.pone.0351395.r001

**Authors:** Manuela Mendonça Figueirêdo Coelho

2 Feb 2026

PONE-D-25-67906

Quality of follow-up care for premature infants in primary health care: A cross-sectional study in Paraná state, Brazil

PLOS One

Dear Dr. Simionato de Assis,

Thank you for submitting your manuscript to PLOS ONE. After careful consideration, we feel that it has merit but does not fully meet PLOS ONE’s publication criteria as it currently stands. Therefore, we invite you to submit a revised version of the manuscript that addresses the points raised during the review process.

==============================

Dear Authors,

After a thorough editorial assessment and peer review by three independent reviewers, your manuscript has been carefully evaluated. The topic addressed is relevant and has potential contribution to the field of maternal and child health and primary health care. However, the reviewers and the editorial analysis identified several conceptual, methodological, and reporting issues that must be substantially improved before the manuscript can be considered for publication.

Therefore, the editorial decision is: **Major Revision**.

**1. RESPONSE TO REVIEWERS**

You are required to address **all comments and recommendations** provided by the three reviewers in full. A detailed point-by-point response letter must be submitted, clearly indicating how each comment was addressed and specifying the exact location of the corresponding modifications in the revised manuscript.

In particular, the following key aspects highlighted by the reviewers must be fully addressed:

- Clearer articulation of the **novelty and scientific contribution** of the study, emphasizing that this manuscript reports an applied evaluation using a previously validated instrument, rather than a validation study;

- Explicit clarification of the **relationship between this study and previous publications** on the development and validation of the Qualipreterm instrument, including: which publication reported the initial development of the instrument; which publication reported the content validation; confirmation that the version used in this study corresponds to the final validated version;

- Clarification regarding the **timing between instrument validation and data collection**, including ethical approval coverage for the use of the questionnaire in this stage;

- Improvement of the description of the **study setting**, including information on the pediatric population, number of births, number of premature births, and infant mortality rates in the state and, when possible, by macro-region;

- Presentation of participants’ characteristics in a **clear descriptive table**, including the number of nurses from each macro-region;

- Expansion of the discussion regarding the **practical and policy implications** of the findings, with explicit recognition of the limitations inherent to cross-sectional self-reported data;

- Full compliance with the **PLOS ONE Data Availability Policy**, including public deposition of anonymized datasets in an appropriate repository.

All reviewer comments must be carefully considered and adequately incorporated into the revised version.

**2. ADDITIONAL EDITORIAL REQUIREMENTS**

In addition to the reviewers’ comments, the editorial evaluation identified further aspects that require mandatory revision:

**2.1 Conceptual Clarification**

**-**The manuscript must clearly state throughout the text that the study evaluates **perceived quality of follow-up care from the perspective of nurses**, not objective service quality.

**-**Title, abstract, objectives, results, and conclusions must be revised to consistently reflect this perspective.

**-**Statements that imply direct assessment of services (e.g., “services were inadequate”) must be reformulated to indicate that these classifications represent nurses’ perceptions.

**2.2 Unit of Analysis and Sampling Procedures**

**-**The manuscript must explicitly clarify that the **unit of analysis is the individual nurse respondent**, not the health service.

**-**Provide detailed methodological information on: how it was ensured that only **one response per health unit** was included; procedures adopted to avoid duplicate responses; how potential multiple responses from the same unit were handled.

**-**Expand the description of recruitment procedures to minimize concerns regarding convenience sampling and potential selection bias.

**2.3 Statistical Analysis**

**-**The authors are strongly encouraged to include additional statistical analyses, such as multivariable models (e.g., logistic or ordinal regression), to explore potential confounders including professional experience, type of unit, urban/rural location, and macro-region.

**-**If such analyses are not conducted, a clear and justified methodological rationale must be provided in the manuscript.

**-**Include **internal consistency analysis** of the Qualipreterm instrument in the current sample (e.g., Cronbach’s alpha for each domain).

**2.4 Spatial Analysis**

**-**Revise the terminology used to describe spatial methods, replacing expressions such as “spatial analysis” with “spatial visualization” where appropriate.

**-**Clearly acknowledge that the maps are descriptive and do not represent inferential spatial modeling.

**2.5 Discussion of Limitations**

**-**Expand the limitations section to explicitly address: the self-reported nature of the data; potential social desirability bias; the response rate and related selection bias; limitations inherent to the cross-sectional design.

**3. DATA AVAILABILITY**

Compliance with the PLOS ONE Data Availability Policy is mandatory. Authors must:

- Deposit the anonymized dataset and relevant materials in a public repository (e.g., OSF, Zenodo, Figshare);

- Update the Data Availability Statement with a permanent link (DOI) to the deposited files;

- If any restrictions apply, provide a detailed justification and description of which variables cannot be shared and why.

**4. RESUBMISSION INSTRUCTIONS**

For resubmission, please provide:

- A comprehensive **point-by-point response letter** addressing all reviewer and editorial comments;

- A revised manuscript with **tracked changes** clearly indicating all modifications;

- Confirmation of data deposition and updated Data Availability Statement.

Please note that full compliance with the requirements listed above is essential for further consideration. Failure to adequately address these points may result in rejection of the manuscript.

We appreciate your submission to the journal and look forward to receiving your revised version.

Sincerely,

**Manuela Coelho**

Academic Editor

PLOS ONE

==============================

Please submit your revised manuscript by Mar 19 2026 11:59PM. If you will need more time than this to complete your revisions, please reply to this message or contact the journal office at plosone@plos.org. When you're ready to submit your revision, log on to https://www.editorialmanager.com/pone/ and select the 'Submissions Needing Revision' folder to locate your manuscript file.

Please include the following items when submitting your revised manuscript:

- A letter that responds to each point raised by the academic editor and reviewer(s). You should upload this letter as a separate file labeled 'Response to Reviewers'.
- A marked-up copy of your manuscript that highlights changes made to the original version. You should upload this as a separate file labeled 'Revised Manuscript with Track Changes'.
- An unmarked version of your revised paper without tracked changes. You should upload this as a separate file labeled 'Manuscript'.

If you would like to make changes to your financial disclosure, please include your updated statement in your cover letter. Guidelines for resubmitting your figure files are available below the reviewer comments at the end of this letter.

If applicable, we recommend that you deposit your laboratory protocols in protocols.io to enhance the reproducibility of your results. Protocols.io assigns your protocol its own identifier (DOI) so that it can be cited independently in the future. For instructions see: https://journals.plos.org/plosone/s/submission-guidelines#loc-laboratory-protocols. Additionally, PLOS ONE offers an option for publishing peer-reviewed Lab Protocol articles, which describe protocols hosted on protocols.io. Read more information on sharing protocols at https://plos.org/protocols?utm_medium=editorial-email&utm_source=authorletters&utm_campaign=protocols.

We look forward to receiving your revised manuscript.

Kind regards,

Manuela Mendonça Figueirêdo Coelho, Ph.D

Academic Editor

PLOS One

Journal Requirements:

When submitting your revision, we need you to address these additional requirements.

1. Please ensure that your manuscript meets PLOS ONE's style requirements, including those for file naming. The PLOS ONE style templates can be found at

https://journals.plos.org/plosone/s/file?id=wjVg/PLOSOne_formatting_sample_main_body.pdf and

https://journals.plos.org/plosone/s/file?id=ba62/PLOSOne_formatting_sample_title_authors_affiliations.pdf

2. Thank you for stating the following in the Acknowledgments Section of your manuscript:

“We acknowledge the Coordenação de Aperfeiçoamento de Pessoal de Nível Superior - Brasil (CAPES) for financial support.”

We note that you have provided funding information that is not currently declared in your Funding Statement. However, funding information should not appear in the Acknowledgments section or other areas of your manuscript. We will only publish funding information present in the Funding Statement section of the online submission form.

Please remove any funding-related text from the manuscript and let us know how you would like to update your Funding Statement. Currently, your Funding Statement reads as follows:

“Coordenação de Aperfeiçoamento de Pessoal de Nível Superior (CAPES)”

Please include your amended statements within your cover letter; we will change the online submission form on your behalf.

3. We note that the grant information you provided in the ‘Funding Information’ and ‘Financial Disclosure’ sections do not match.

When you resubmit, please ensure that you provide the correct grant numbers for the awards you received for your study in the ‘Funding Information’ section.

4. Thank you for stating the following financial disclosure:

“Coordenação de Aperfeiçoamento de Pessoal de Nível Superior (CAPES)”

Please state what role the funders took in the study.  If the funders had no role, please state: "The funders had no role in study design, data collection and analysis, decision to publish, or preparation of the manuscript."

If this statement is not correct you must amend it as needed.

Please include this amended Role of Funder statement in your cover letter; we will change the online submission form on your behalf.

5. We note that you have indicated that there are restrictions to data sharing for this study. PLOS only allows data to be available upon request if there are legal or ethical restrictions on sharing data publicly. For more information on unacceptable data access restrictions, please see http://journals.plos.org/plosone/s/data-availability#loc-unacceptable-data-access-restrictions.

Before we proceed with your manuscript, please address the following prompts:

a) If there are ethical or legal restrictions on sharing a de-identified data set, please explain them in detail (e.g., data contain potentially identifying or sensitive patient information, data are owned by a third-party organization, etc.) and who has imposed them (e.g., a Research Ethics Committee or Institutional Review Board, etc.). Please also provide contact information for a data access committee, ethics committee, or other institutional body to which data requests may be sent.

b) If there are no restrictions, please upload the minimal anonymized data set necessary to replicate your study findings to a stable, public repository and provide us with the relevant URLs, DOIs, or accession numbers. For a list of recommended repositories, please see

https://journals.plos.org/plosone/s/recommended-repositories. You also have the option of uploading the data as Supporting Information files, but we would recommend depositing data directly to a data repository if possible.

We will update your Data Availability statement on your behalf to reflect the information you provide.

6. In the online submission form you indicate that your data is not available for proprietary reasons and have provided a contact point for accessing this data. Please note that your current contact point is a co-author on this manuscript. According to our Data Policy, the contact point must not be an author on the manuscript and must be an institutional contact, ideally not an individual. Please revise your data statement to a non-author institutional point of contact, such as a data access or ethics committee, and send this to us via return email. Please also include contact information for the third party organization, and please include the full citation of where the data can be found.

7. We note that Figures 1 - 6 in your submission contain map images which may be copyrighted. All PLOS content is published under the Creative Commons Attribution License (CC BY 4.0), which means that the manuscript, images, and Supporting Information files will be freely available online, and any third party is permitted to access, download, copy, distribute, and use these materials in any way, even commercially, with proper attribution. For these reasons, we cannot publish previously copyrighted maps or satellite images created using proprietary data, such as Google software (Google Maps, Street View, and Earth). For more information, see our copyright guidelines: http://journals.plos.org/plosone/s/licenses-and-copyright.

We require you to either (1) present written permission from the copyright holder to publish these figures specifically under the CC BY 4.0 license, or (2) remove the figures from your submission:

1. You may seek permission from the original copyright holder of Figure(s) [#] to publish the content specifically under the CC BY 4.0 license.

We recommend that you contact the original copyright holder with the Content Permission Form (http://journals.plos.org/plosone/s/file?id=7c09/content-permission-form.pdf) and the following text:

“I request permission for the open-access journal PLOS ONE to publish XXX under the Creative Commons Attribution License (CCAL) CC BY 4.0 (http://creativecommons.org/licenses/by/4.0/). Please be aware that this license allows unrestricted use and distribution, even commercially, by third parties. Please reply and provide explicit written permission to publish XXX under a CC BY license and complete the attached form.”

Please upload the completed Content Permission Form or other proof of granted permissions as an "Other" file with your submission.

In the figure caption of the copyrighted figure, please include the following text: “Reprinted from [ref] under a CC BY license, with permission from [name of publisher], original copyright [original copyright year].”

2. If you are unable to obtain permission from the original copyright holder to publish these figures under the CC BY 4.0 license or if the copyright holder’s requirements are incompatible with the CC BY 4.0 license, please either i) remove the figure or ii) supply a replacement figure that complies with the CC BY 4.0 license. Please check copyright information on all replacement figures and update the figure caption with source information. If applicable, please specify in the figure caption text when a figure is similar but not identical to the original image and is therefore for illustrative purposes only.

The following resources for replacing copyrighted map figures may be helpful:

USGS National Map Viewer (public domain): http://viewer.nationalmap.gov/viewer/

The Gateway to Astronaut Photography of Earth (public domain): http://eol.jsc.nasa.gov/sseop/clickmap/

Maps at the CIA (public domain): https://www.cia.gov/library/publications/the-world-factbook/index.html and https://www.cia.gov/library/publications/cia-maps-publications/index.html

NASA Earth Observatory (public domain): http://earthobservatory.nasa.gov/

Landsat: http://landsat.visibleearth.nasa.gov/

USGS EROS (Earth Resources Observatory and Science (EROS) Center) (public domain): http://eros.usgs.gov/#

Natural Earth (public domain): http://www.naturalearthdata.com/

8. If the reviewer comments include a recommendation to cite specific previously published works, please review and evaluate these publications to determine whether they are relevant and should be cited. There is no requirement to cite these works unless the editor has indicated otherwise.

[Note: HTML markup is below. Please do not edit.]

Reviewers' comments:

Reviewer's Responses to Questions

**Comments to the Author**

1. Is the manuscript technically sound, and do the data support the conclusions?

The manuscript must describe a technically sound piece of scientific research with data that supports the conclusions. Experiments must have been conducted rigorously, with appropriate controls, replication, and sample sizes. The conclusions must be drawn appropriately based on the data presented.

Reviewer #1: Yes

Reviewer #2: No

Reviewer #3: Yes

**********

2. Has the statistical analysis been performed appropriately and rigorously?

Reviewer #1: I Don't Know

Reviewer #2: No

Reviewer #3: Yes

**********

3. Have the authors made all data underlying the findings in their manuscript fully available?

The PLOS Data policy requires authors to make all data underlying the findings described in their manuscript fully available without restriction, with rare exception (please refer to the Data Availability Statement in the manuscript PDF file). The data should be provided as part of the manuscript or its supporting information, or deposited to a public repository. For example, in addition to summary statistics, the data points behind means, medians and variance measures should be available. If there are restrictions on publicly sharing data—e.g. participant privacy or use of data from a third party—those must be specified.

Reviewer #1: Yes

Reviewer #2: No

Reviewer #3: No

**********

4. Is the manuscript presented in an intelligible fashion and written in standard English?

PLOS ONE does not copyedit accepted manuscripts, so the language in submitted articles must be clear, correct, and unambiguous. Any typographical or grammatical errors should be corrected at revision, so please note any specific errors here.

Reviewer #1: Yes

Reviewer #2: Yes

Reviewer #3: Yes

**********

5. Review Comments to the Author

Please use the space provided to explain your answers to the questions above. You may also include additional comments for the author, including concerns about dual publication, research ethics, or publication ethics. (Please upload your review as an attachment if it exceeds 20,000 characters)

Reviewer #1: To the Authors

This paper addresses an important yet understudied issue: the quality of follow-up care for preterm infants in primary health care settings. It evaluates this issue from the nurses' perspective using the Qualipreterm rating scale. This study, based on a large sample size at the state level, may provide valuable insights for healthcare settings and healthcare system organizations. However, as you note, while it may be useful for national health policy recommendations, caution is warranted in its non-national findings.

The following points should be considered to strengthen this paper.

1. Clarifying Novelty and Contribution

While this study clearly involves original data collection and analysis, the paper does not clearly explain its novelty.

The value of this study lies not in the development or validation of a new rating scale, but in the application of a validated tool to evaluate actual clinical practice on a large scale. Please consider revising the introduction and discussion to more clearly state the following points: Since there was another paper published in the same year by the same research group, with similar keywords and authors, we also checked for the possibility of duplicate submission.

As a result, the previous paper demonstrated that the content validity of the Qualipreterm guide was verified (Delphi method) to assess the "quality of follow-up care for preterm infants in PHC." This paper uses Qualipreterm to cross-sectionally measure the quality of follow-up care in Paraná State from the perspective of PHC nurses and analyze regional differences. Therefore, we understand that the objectives of this paper are different: one is to develop and refine a tool, while the other is to measure the current situation using a tool.

With this in mind, this paper aims to:

• What new insights can be gained by applying the Qualipreterm assessment scale in everyday primary health care settings?

• How do the findings of this study deepen our understanding of weaknesses and variations in follow-up care for preterm infants?

• Why are these results relevant beyond specific regional contexts?

More clearly articulating this contribution would significantly increase the value of the manuscript.

2. Relationship to the Development and Validation of Previous Assessment Tools

This paper would benefit from a clearer explanation of the relationship between this study and previous publications reporting the development and validation of the Qualipreterm assessment tool.

Please clarify the following:

• Which publication reported the initial development of the Qualipreterm guide?

• Which publication reported the content validation of the assessment tool?

• Did the version of the assessment tool used in this study match the final validated version?

This clarification is important for transparency and helps readers understand the methodological background of this study.

3. Timing of Use of the Assessment Tool and Ethical Considerations

The application assessment reported in this manuscript appears to have been conducted close to or overlapping with the assessment tool validation process.

While this is not inherently an ethical issue, the manuscript should clearly state the following:

• How was the timing of validation and application managed?

• Did ethical approval and participant information cover the use of the questionnaire at that stage?

• How were potential limitations related to the maturity of the questionnaire considered in interpreting the results?

Addressing these points will strengthen the methodological soundness of this study.

4. Interpretation and Implications

The identification of weaknesses in specific areas (particularly discharge planning) and the observed regional variations are potentially important findings. However, the discussion could be strengthened by:

• Deeper consideration of possible system-level or organizational-level explanations.

• Clearer articulation of practical or policy implications.

• Explicit recognition of the limitations of nurse-reported cross-sectional data.

Summary Recommendations

This paper presents a potentially valuable practice-based evaluation using original data and appropriate analyses. We hope that by more clearly articulating its novelty, increasing transparency regarding the questionnaire, and strengthening interpretation, this paper will make a meaningful contribution to the literature.

Sincerely,

From one of the reviewers

Reviewer #2: This manuscript addresses a relevant topic; however, it presents fundamental conceptual and methodological flaws that undermine the validity of the findings.

First, the study claims to assess the “quality of follow-up care,” yet relies exclusively on self-reported perceptions of nurses. Perceived quality cannot be equated with actual quality of care in the absence of objective indicators, clinical outcomes, service audits, or triangulation with other data sources. This represents a core misclassification of the primary outcome rather than a minor limitation.

Second, the response rate (~17%) is critically low for a state-level cross-sectional study. No comparison between respondents and non-respondents is provided, making it impossible to assess representativeness. The voluntary, online recruitment strategy introduces substantial risk of selection bias, severely limiting generalizability.

Third, the unit of analysis is unclear. Although nurses provided individual responses, the analyses and interpretations implicitly treat the data as representative of health services. The manuscript does not clarify how nurses were selected in units with multiple professionals, nor whether multiple responses per unit occurred, resulting in a unit-of-analysis error.

Fourth, the statistical analysis is insufficient. The exclusive use of descriptive statistics and chi-square tests, without multivariable modeling or adjustment for confounders (e.g., professional experience, unit characteristics, urban/rural location), does not support the conclusions regarding regional differences or heterogeneity in care quality.

Fifth, the spatial analysis is purely descriptive. No spatial statistical methods were applied, and the maps do not add analytical value beyond tabulated results.

Finally, the manuscript does not comply with PLOS ONE’s Data Availability Policy. Stating that data are available “upon reasonable request” due to ethical restrictions is insufficient for anonymized survey data of health professionals, and no justification is provided regarding which variables cannot be shared or why anonymization is not feasible.

These limitations are fundamental and cannot be resolved through revision of the current manuscript. Addressing them would require a redesigned study with a different methodological framework. Therefore, I do not believe this manuscript is suitable for publication in PLOS ONE.

Reviewer #3: General comments:

Simionato et al. analyzed the quality of follow-up programs for premature infants in a Brazilian state from the perspective of primary healthcare nurses, discussing the need for multifaceted improvements.

The topic is important; however, the manuscript requires some minor revisions to be considered acceptable.

Minor points:

1. The authors should provide an explanation of the pediatric population, the number of births, the number of premature births, and the premature infant mortality rate for the entire state, as well as for each region, in the "Study Setting" section.

2. Lines 263-271: The background information of the primary healthcare nurses who participated in the study would be better presented in a table. Additionally, the number of nurses who participated from each of the four regions should also be presented in the results.

3. Is there any common educational system for primary healthcare nurses regarding follow-up care for premature infants at the national or state level? If so, please mention it in the manuscript.

**********

6. PLOS authors have the option to publish the peer review history of their article (what does this mean?). If published, this will include your full peer review and any attached files.

If you choose “no”, your identity will remain anonymous but your review may still be made public.

**Do you want your identity to be public for this peer review?** For information about this choice, including consent withdrawal, please see our Privacy Policy.

Reviewer #1: No

Reviewer #2: No

Reviewer #3: No

**********

[NOTE: If reviewer comments were submitted as an attachment file, they will be attached to this email and accessible via the submission site. Please log into your account, locate the manuscript record, and check for the action link "View Attachments". If this link does not appear, there are no attachment files.]

To ensure your figures meet our technical requirements, please review our figure guidelines: https://journals.plos.org/plosone/s/figures

You may also use PLOS’s free figure tool, NAAS, to help you prepare publication quality figures: https://journals.plos.org/plosone/s/figures#loc-tools-for-figure-preparation.

NAAS will assess whether your figures meet our technical requirements by comparing each figure against our figure specifications.

# Author comment: Author response to Decision Letter 1

**DOI:** 10.1371/journal.pone.0351395.r002

16 May 2026

Dear Editor,

Thank you for your feedback. I have addressed both issues:

1. File inventory: I have verified that all files are current and correct.

2. Funding information: I have added a "Funding" section to the manuscript (between Acknowledgments and Data Availability Statement) to match the funding information provided in the system. Both sections now state:

"This work was supported by the Coordination for the Improvement of Higher Education Personnel - Brazil (CAPES) - Grant Number 88887.691385/2022-00."

Thank you for your consideration.

Best regards,

Ivaneliza Simionato de Assis

Attachment Submitted filename: Response_Letter.docx

# Editor report: Decision Letter 1

**DOI:** 10.1371/journal.pone.0351395.r003

**Authors:** Manuela Mendonça Figueirêdo Coelho

28 May 2026

Quality of follow-up care for premature infants in primary health care: A cross-sectional study from nurses' perspective in Paraná state, Brazil

PONE-D-25-67906R1

Dear Dr. Simionato de Assis,

We’re pleased to inform you that your manuscript has been judged scientifically suitable for publication and will be formally accepted for publication once it meets all outstanding technical requirements.

Within one week, you’ll receive an e-mail detailing the required amendments. When these have been addressed, you’ll receive a formal acceptance letter and your manuscript will be scheduled for publication.

An invoice will be generated when your article is formally accepted. Please note, if your institution has a publishing partnership with PLOS and your article meets the relevant criteria, all or part of your publication costs will be covered. Please make sure your user information is up-to-date by logging into Editorial Manager at Editorial Manager® and clicking the ‘Update My Information' link at the top of the page. For questions related to billing, please contact billing support.

If your institution or institutions have a press office, please notify them about your upcoming paper to help maximize its impact. If they’ll be preparing press materials, please inform our press team as soon as possible -- no later than 48 hours after receiving the formal acceptance. Your manuscript will remain under strict press embargo until 2 pm Eastern Time on the date of publication. For more information, please contact onepress@plos.org.

Kind regards,

Manuela Mendonça Figueirêdo Coelho, Ph.D

Academic Editor

PLOS One

Additional Editor Comments (optional):

Reviewers' comments:

# Editor report: Acceptance letter

**DOI:** 10.1371/journal.pone.0351395.r004

**Authors:** Manuela Mendonça Figueirêdo Coelho

PONE-D-25-67906R1

PLOS One

Dear Dr. Simionato de Assis,

I'm pleased to inform you that your manuscript has been deemed suitable for publication in PLOS One. Congratulations! Your manuscript is now being handed over to our production team.

At this stage, our production department will prepare your paper for publication. This includes ensuring the following:

* All references, tables, and figures are properly cited

* All relevant supporting information is included in the manuscript submission,

* There are no issues that prevent the paper from being properly typeset

You will receive further instructions from the production team, including instructions on how to review your proof when it is ready. Please keep in mind that we are working through a large volume of accepted articles, so please give us a few days to review your paper and let you know the next and final steps.

Lastly, if your institution or institutions have a press office, please let them know about your upcoming paper now to help maximize its impact. If they'll be preparing press materials, please inform our press team within the next 48 hours. Your manuscript will remain under strict press embargo until 2 pm Eastern Time on the date of publication. For more information, please contact onepress@plos.org.

You will receive an invoice from PLOS for your publication fee after your manuscript has reached the completed accept phase. If you receive an email requesting payment before acceptance or for any other service, this may be a phishing scheme. Learn how to identify phishing emails and protect your accounts at https://explore.plos.org/phishing.

If we can help with anything else, please email us at customercare@plos.org.

Thank you for submitting your work to PLOS ONE and supporting open access.

Kind regards,

PLOS ONE Editorial Office Staff

on behalf of

Dr. Manuela Mendonça Figueirêdo Coelho

Academic Editor

PLOS One
